# A New Cache Update Scheme Using Reinforcement Learning for Coded Video Streaming Systems

**DOI:** 10.3390/s21082867

**Published:** 2021-04-19

**Authors:** Yu-Sin Kim, Jeong-Min Lee, Jong-Yeol Ryu, Tae-Won Ban

**Affiliations:** 1 Algorithm Team, Carvi, Seoul 08513, Korea; usin1216@carvi.co.kr; 2 Department of Information and Communication Engineering, Gyeongsang National University, Gyeongnam 53064, Korea; ljmin200002@gmail.com (J.-M.L.); jongyeol_ryu@gnu.ac.kr (J.-Y.R.)

**Keywords:** streaming, multimedia, reinforcement learning, cache, exclusive OR

## Abstract

As the demand for video streaming has been rapidly increasing recently, new technologies for improving the efficiency of video streaming have attracted much attention. In this paper, we thus investigate how to improve the efficiency of video streaming by using clients’ cache storage considering exclusive OR (XOR) coding-based video streaming where multiple different video contents can be simultaneously transmitted in one transmission as long as prerequisite conditions are satisfied, and the efficiency of video streaming can be thus significantly enhanced. We also propose a new cache update scheme using reinforcement learning. The proposed scheme uses a *K*-actor-critic (*K*-AC) network that can mitigate the disadvantage of actor-critic networks by yielding *K* candidate outputs and by selecting the final output with the highest value out of the *K* candidates. The *K*-AC exists in each client, and each client can train it by using only locally available information without any feedback or signaling so that the proposed cache update scheme is a completely decentralized scheme. The performance of the proposed cache update scheme was analyzed in terms of the average number of transmissions for XOR coding-based video streaming and was compared to that of conventional cache update schemes. Our numerical results show that the proposed cache update scheme can reduce the number of transmissions up to 24% when the number of videos is 100, the number of clients is 50, and the cache size is 5.

## 1. Introduction

In recent years, Internet traffic has been rapidly increasing and is expected to increase more rapidly in the future [1,2]. In particular, it is also expected that video streaming traffic will account for 82% of the global Internet traffic by 2022 due to the wide popularity of various video streaming platforms such as YouTube [1]. This trend is more pronounced in mobile networks, and many advanced techniques have been thus investigated to increase the capacity of next-generation mobile communication networks [3,4,5]. Along with many technologies to increase network capacity by using a wide bandwidth or by increasing spectral efficiency, other technologies for reducing network traffic are also attracting much attention as another alternative [6,7]. Multicast (MC) transmission can reduce network traffic by transmitting a video to multiple clients in one transmission if the clients requested the same video at the same time [6]. Proxy servers with cache can significantly reduce network traffic, and bandwidth optimization for real-time video traffic transmission through a proxy server was investigated in [7]. In particular, MC-aware caching can better exploit the available cache space and can yield a gain of 19% over existing caching schemes [6]. Many studies have studied how to reduce network traffic by using the transmitters’ cache storage, while the low cost and large capacity of storage motivated some studies to focus on the clients’ cache storage [8,9,10,11,12,13]. In this paper, we thus investigate a new video streaming system using clients’ cache and XOR-based index coding. In the new video streaming system, multiple different video contents can be transmitted in one transmission if prerequisites are satisfied, and transmission efficiency can be thus significantly improved. Cache update is an important factor in video streaming systems [14,15,16,17,18,19]. However, there have been no previous studies that investigated cache update policies for the index coding-based video streaming system. Thus, we investigate how to update the clients’ cache for index coding-based video streaming systems in order to use the clients’ cache more efficiently, and we propose a new cache update scheme for clients using deep reinforcement learning. The proposed cache update scheme was based on a new architecture called *K*-actor-critic (*K*-AC) that can mitigate the shortcomings of the actor-critic (AC) network architecture. The *K*-AC network that consists of an actor network and the main value network exists in each client, and each client can thus update its own cache in a fully decentralized manner without any exchange of information or signaling. In this work, we assumed that all clients have different popularity for videos, and the popularity for each client is time varying, contrary to most conventional studies assuming that video popularity is the same for all clients and is time invariant.

The rest of this paper is organized as follows. We investigate related studies in Section 2. Section 3 introduces the system model considered in this paper and describes the basic concept of XOR coding-based video streaming. A mathematical ground for reducing the number of XOR operations is also introduced in Section 3. In Section 4, we propose a new cache update algorithm using reinforcement learning for index coding-based video streaming systems. Section 5 shows the numerical results. Finally, this paper is concluded in Section 6.

## 2. Related Work

Contrary to conventional strategies that used the transmitters’ cache, there have been recent studies to exploit the clients’ cache storage [8,9,10,11,12,13]. Methods that can efficiently exploit the clients’ cache storage were investigated from the viewpoint of information theory [8,9,10]. Lower and upper bounds were presented on the capacity-memory tradeoff of an erasure broadcast network with two disjoint sets of receivers: a set of weak receivers with equal erasure probabilities and equal cache sizes and a set of strong receivers with equal erasure probabilities and no cache memories [8]. It was proposed to exploit the limited cache packets as side information to cancel incoming interference at the receiver side by considering a stochastic network [9]. A new inner bound on the capacity region of the general index coding problem was investigated by relying on a random coding scheme and optimal decoding [10]. A new concept using index coding for transmitting contents was proposed in [11], where multiple contents were index coded, and they can be transmitted in one transmission over a single channel if some prerequisites are satisfied. A new algorithm of the index code and time resource allocation that can minimize wireless transmission outage probability with a low complexity was proposed [12]. Many studies focusing on the clients’ cache mainly investigated theoretical performance analysis or optimal index code design by considering simplistic or unrealistic system models, while the index code was applied to a realistic system in [13]. Exclusive OR (XOR)-based index coding can be applied to large-scale video streaming systems while providing a complete backward compatibility with existing streaming schemes such as unicast (UC) and MC thanks to the properties of the XOR operator such as zero-identity, self-inverse, commutativity, and associativity [13].

On the other hand, there have been many studies on cache update [14,15,16,17,18,19]. The performance of FIFO, the least recently used (LRU), and the least frequently used (LFU) schemes was analyzed in terms of the rate at which a particular request is returned before a given deadline [14] and in terms of hit rate [15]. A novel content-aware cache replacement algorithm taking advantage of content demand forecasts was investigated to efficiently use limited caches in size [16]. LRU-K, which is a combination of LRU and LFU, was proposed [17]. They simulated TV distribution with time-shift and investigated the effect of introducing a local cache close to the viewers and what impact TV program popularity, program set size, cache replacement policy, and other factors had on the caching efficiency [18]. A new concept that cache servers share request information to predict the popularity of contents through regression was proposed [19]. A deep Q-network (DQN)-based cache update scheme for edge cache networks was proposed [20]. They aimed at maximizing the overall quality of 360${}^{{}^{\circ}}$ videos delivered to the end-users by caching the most popular ones at base quality along with a virtual viewport in high quality. A new centralized cache update scheme using the Wolpertinger architecture for base stations was proposed [21]. The Wolpertinger architecture selects a single proto-action from the actor network and selects the *K*-closest action around the proto-action for the input of the critic network [22]. Contrary to the Wolpertinger architecture, our *K*-AC directly selects *K* candidate actions with the highest *Q* values from the actor network for the input of the critic network, inspired by the fact that the actions in our problem do not have a strong correlation with each other. Despite these many existing studies on cache update, the simplest cache update scheme, first-in first-out (FIFO), was only considered in index coding-based video streaming systems [13], and there have been no cache update schemes targeting index coding-based video streaming systems. In index coding-based video streaming systems, each client needs to update its cache so as to increase the probability of index coding with other clients, as well as its own hit probability, contrary to conventional video streaming systems where each client’s hit probability is only considered.

## 3. XOR Coding-Based Streaming System

**Figure 1:**
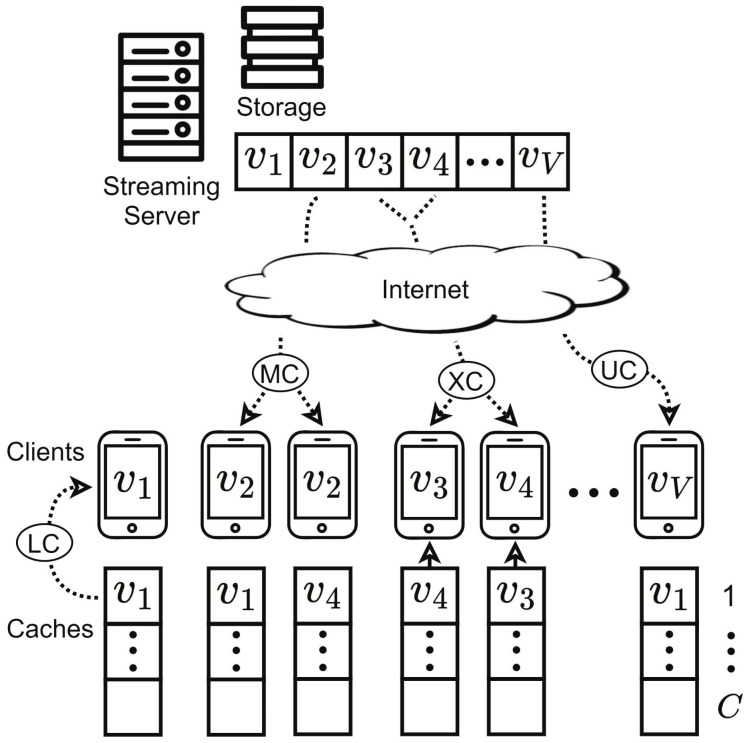
System architecture.

We investigated a coded video streaming system, as depicted in Figure 1, which consisted of *N* clients and a streaming server. All the clients and the server were equipped with cache. Clients’ cache can store *C* videos, while the server’s cache can store *V* videos (V≫C). It was assumed that all videos had the same length in time. Even if multiple clients request different videos, they can be selectively XOR-encoded into one bit stream according to the status of their caches [13]. For a given set of clients, if every client in the set has all videos requested by the remaining clients in its cache, then all the clients in the set can receive their videos through XOR coding in one transmission. This is called XOR-cast (XC). The XOR-encoded bit stream is transmitted to the clients by one transmission, and we can reduce the number of transmissions for the videos requested by the clients. Then, each client restores its video by decoding the received bit stream with the contents stored in its cache [13]. As a specific example, the client requesting $v_{1}$ in Figure 1 plays the video $v_{1}$ stored in its cache without receiving any data from the server, which is called local cast (LC). The two clients requesting $v_{2}$ can stream $v_{2}$ from the same channel through MC. The client requesting $v_{3}$ and the client requesting $v_{4}$ store $v_{4}$ and $v_{3}$, respectively, and the server thus XOR encodes $v_{3}$ and $v_{4}$. $\left(v_{3}⊕v_{4}\right)$ is transmitted over a single channel through XC even though $v_{3}$ and $v_{4}$ are different. The client that requested $v_{3}$ restores $v_{3}$ by using $\left(v_{3}⊕v_{4}\right)⊕v_{4}=v_{3}$, and the client that requested $v_{4}$ restores $v_{4}$ by using $\left(v_{3}⊕v_{4}\right)⊕v_{3}=v_{4}$, where the equalities are valid due to the properties of the XOR operator such as zero-identity, self-inverse, commutativity, and associativity.

The relative popularity of the *v*-th most popular one among *V* videos is modeled by the Zipf distribution, which is given by:

(1) $$f\left(v;\beta ,V\right)=\frac{1/v^{\beta }}{\sum_{k=1}^{V}\left(1/k^{\beta }\right)},$$

where β is the Zipf parameter characterizing the distribution and $\sum_{v=1}^{V}f\left(v;\beta ,V\right)=1$ regardless of β [23]. Contrary to most conventional studies that assumed that all clients have the same relative popularity for all videos and the relative popularity is time-invariant, we assumed that all clients have different popularity and that the popularity for each client is time varying. Client *n* requests a video *v* at time *t* with a probability $P_{\left(n,v\right)}^{t}$. $P_{\left(n,v\right)}^{t}$’s are time varying and different for all clients and can be defined as:

(2) $$P_{\left(n,v\right)}^{t+1}=\left\{\begin{array}{cc} \rho P_{\left(n,v\right)}^{t}+\left(1-\rho \right)f\left(w;\beta ,V\right) & \mathrm{with}\;\mathrm{prob}.\;p\\ P_{\left(n,v\right)}^{t} & \mathrm{with}\;\mathrm{prob}.\;1-p, \end{array}\right.$$

where *p* denotes the probability that the rank *v* of a video changes to a new rank *w* for the client *n*, *w* denotes that the new rank of the video *v* is a random integer between one and *V*, and ρ denotes a correlation between the old rank *v* and the new rank *w* satisfying 0<ρ<1 for all v∈{1,…,V}. The initial probability of $P_{\left(n,v\right)}^{t}$ is given by $P_{\left(n,v\right)}^{0}=f\left(v;\beta ,V\right)$. *p* and ρ can adjust the frequency and the amount of change in popularity for video *v*, respectively.

**Figure 2:**
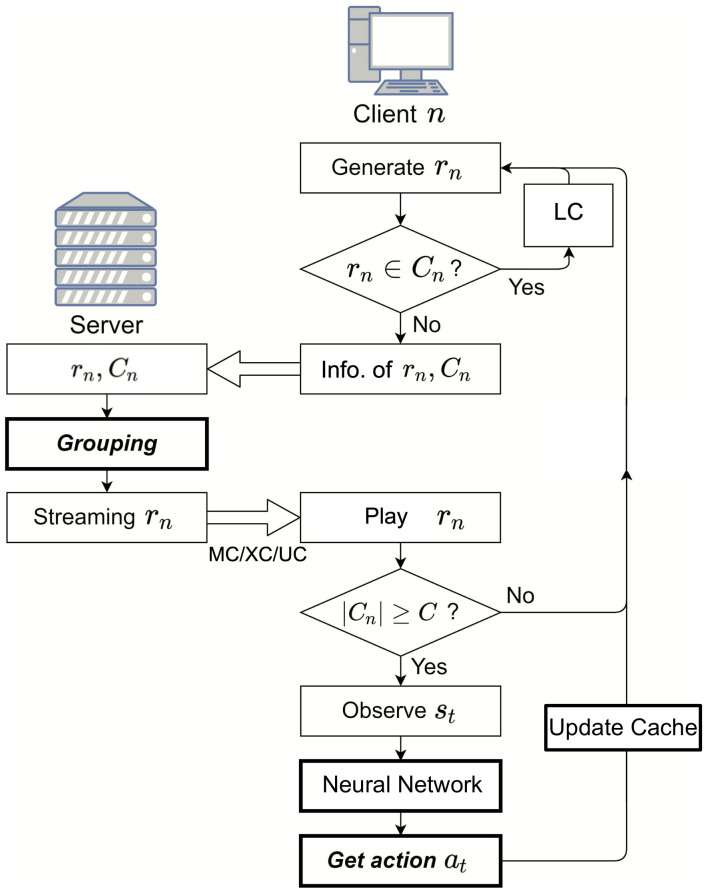
Overall procedures of XOR coding-based streaming.

Figure 2 shows the overall procedure of XOR coding-based streaming systems. $r_{n}$ and $C_{n}$ denote a video that client *n* requests and the set of videos stored in the cache of the client *n*, respectively. $|C_{n}|=C$, where $|\dot{|}$ denotes the cardinality of a set. In this system, we aimed to reduce the number of transmissions required to transmit the *N* videos $\left\{r_{n}|n\in U\right\}$ requested by the *N* clients, where U denotes the set of the whole clients and is given by U={1,2,…,N}. If $r_{n}\in C_{n}$, which denotes that $r_{n}$ is stored in the client *n*’s cache, then the client *n* can play the $r_{n}$ stored in the cache through LC without connecting to the server. The set of clients who can play a video through LC can be found as:

(3) $$\begin{array}{c} G_{\mathrm{LC}}=\left\{n|r_{n}\in C_{n},\;n\in U\right\}. \end{array}$$

If an arbitrary client *n* is not included in $G_{\mathrm{LC}}$, it transmits a request message including the information of $r_{n}$ and $C_{n}$ to the server. The extra overhead per client required to send $C_{n}$, denoted by O, can be calculated as:

(4) $$O=\lceil \log_{2}V\rceil \times C,$$

where ⌈·⌉ denotes the ceiling function. O is linearly proportional to *C*, which is not a big value in real environments and is logarithmically proportional to *V*. In addition, O is ignorable, compared to the size of recent video contents. If there exist multiple clients that have requested the same video, they can all receive the video through MC in one transmission. The set of clients who can receive a video through MC can be found as:

(5) $$\begin{array}{c} \begin{array}{c} G_{\mathrm{MC}}=\left\{n\;|\;\left|\left\{i|r_{i}=r_{n},\left(i\neq n\right)\&\left(i\in U\backslash G_{\mathrm{LC}}\right)\right\}\right|\geq 1,n\in U\backslash G_{\mathrm{LC}}\right\}, \end{array} \end{array}$$

where A\B denotes the set difference of sets *A* and *B*. $G_{\mathrm{MC}}$ includes all clients that can receive a video through MC, and the number of transmissions required for $G_{\mathrm{MC}}$ denoted by $K_{\mathrm{MC}}$ can be calculated by:

(6) $$\begin{array}{c} K_{\mathrm{MC}}=\left|\sum_{n\in G_{\mathrm{MC}}}\left\{r_{n}\right\}\right|, \end{array}$$

where (A+B) denotes the union of two sets A and B, removing duplicate elements instead of the arithmetic addition for notational simplicity. Then, all remaining clients that are not included in $G_{\mathrm{LC}}$ or $G_{\mathrm{MC}}$, given by $X=U\backslash G_{\mathrm{LC}}\backslash G_{\mathrm{MC}}$, become candidates for XC, and the server sorts out the clients eligible for XC. A client i∈X can receive a video content through XC together with other clients in X that satisfy $\left\{j|r_{i}\in C_{j},r_{j}\in C_{i},j\neq i,j\in X\right\}$. They compose one group for XC, and the server XOR encodes their video contents into one bit stream and transmits the bit stream in one transmission. For each client *i* in X, the server looks for other clients in X that can be grouped with the client *i* for XC, and the result can be obtained by:

(7) $$\begin{array}{c} G_{\mathrm{XC}}\;=\;\left\{\left\{i\right\}\;+\;\left\{j|r_{i}\;\in \;C_{j},r_{j}\;\in \;C_{i},j\neq i,j\in X\right\}|i\;\in \;X\right\}. \end{array}$$

$G_{\mathrm{XC}}$ is a set of sets and $G_{\mathrm{XC}}\left[k\right]$ denotes the *k*-th element of $G_{\mathrm{XC}}$, which is a set. If $G_{\mathrm{XC}}\left[k\right]$ includes a single client, $|G_{\mathrm{XC}}\left[k\right]|=1$, the client will receive the video by UC, and if $|G_{\mathrm{XC}}\left[k\right]|=2$, the two clients will receive their videos by XC with no other options. If $|G_{\mathrm{XC}}\left[k\right]|\geq 3$, the possibility of XC among the rest of the clients except for $G_{\mathrm{XC}}\left[k\right]\left[1\right]$ exists, and there can be thus multiple options that the clients can be grouped for XC. We need to reduce the number of XOR operations, and the number of XOR operations decreases as the cardinalities of XC groups are even, as described in Theorem 1 and Remark 1. The server sorts all groups in $G_{\mathrm{XC}}$ in ascending order according to their cardinalities and saves them in ${\hat{G}}_{\mathrm{XC}}$. ${\hat{G}}_{\mathrm{XC}}\left[\hat{k}\right]$ denotes the group with the *k*-th smallest cardinality, and $\left|{\hat{G}}_{\mathrm{XC}}\left[\hat{k}\right]\right|\leq \left|{\hat{G}}_{\mathrm{XC}}\left[\hat{k+1}\right]\right|$ is thus satisfied for all *k*’s, 1≤k≤|X|−1. Then, XC groups can be obtained by:

(8) $$\begin{array}{c} {\tilde{G}}_{\mathrm{XC}}\left[i\right]={\hat{G}}_{\mathrm{XC}}\left[i\right]\backslash \sum_{j=1}^{i-1}{\hat{G}}_{\mathrm{XC}}\left[j\right], \end{array}$$

where all duplicate groups are removed and smaller XC groups are chosen instead of larger ones when there are multiple options for XC grouping. Finally, the set of clients who can stream a video through XC can be given as:

(9) $$\begin{array}{c} G_{\mathrm{XC}}=\sum_{i=1}^{\left|{\tilde{G}}_{\mathrm{XC}}\right|}{\tilde{G}}_{\mathrm{XC}}\left[i\right], \end{array}$$

and the number of transmissions required for $G_{\mathrm{XC}}$ is denoted by $K_{\mathrm{XC}}$ and can be calculated by:

(10) $$\begin{array}{c} K_{\mathrm{XC}}=\left|{\tilde{G}}_{\mathrm{XC}}\right|. \end{array}$$

As a specific example, assume that $G_{\mathrm{XC}}=\left\{\left\{1,2,3\right\},\left\{2,1,3\right\},\left\{3,1,2,4\right\},\left\{4,3\right\}\right\}$. Then, two different options for making XC groups exist; A:{{1,2},{3,4}}, which requires six XOR operations, and B:{{1,2,3},{4}}, which requires eight XOR operations. Even though the two options both require two transmissions, ${\hat{G}}_{\mathrm{XC}}$ is given as:

(11) $$\begin{array}{cc} {\hat{G}}_{\mathrm{XC}} & =\{\{4,3\},\{1,2,3\},\{2,1,3\},\{3,1,2,4\}\}, \end{array}$$

and ${\tilde{G}}_{\mathrm{XC}}$ is calculated as:

(12) $$\begin{array}{cc} {\tilde{G}}_{\mathrm{XC}} & =\{\{4,3\},\{1,2\}\} \end{array}$$

by (8). Thus, the option *A* with two XC groups {1,2} and {3,4} is chosen instead of the option *B* by (8) due to its smaller number of XOR operations, where six is the minimum number of XOR operations for K=2 and N=4, given by Theorem 1. $G_{\mathrm{XC}}=\left\{1,2,3,4\right\}$.

**Theorem 1.**  *For M XC groups with N clients, the minimum total number of XOR operations required by the server and the clients is $\frac{N^{2}}{M}-M$.*

**Proof.**  For an XC group consisting of *n* clients, the server requires (n−1) XOR operations for encoding, and each client in the XC group also requires (n−1) XOR operations for decoding. Thus, the total number of XOR operations required by the server and the clients can be calculated by $\left(n-1\right)+n\left(n-1\right)=n^{2}-1$. If we have *M* XC groups and *N* clients in total and $N_{k}$ denotes the cardinality of the *i*-th XC group, the total number of XOR operations required by both the server and the clients can be calculated as:

(13) $$\begin{array}{c} O=\sum_{i=1}^{M}\left(N_{i}^{2}-1\right)=\sum_{i=1}^{M}N_{i}^{2}-M, \end{array}$$

where $\sum_{i=1}^{M}N_{i}^{2}$ can be rewritten as $\sum_{i=1}^{M}N_{i}^{2}=M\frac{\sum_{i=1}^{M}N_{i}^{2}}{M}=ME\left[N_{i}^{2}\right]$. For an arbitrary random variable *X*, $V\left[X\right]=E\left[X^{2}\right]-E{\left[X\right]}^{2}$. Thus, (13) can be rewritten as:

(14) $$\begin{array}{ll} O & =M\left(E[N_{i}^{2}]-1\right)\\ & =M\left(E{\left[N_{i}\right]}^{2}+V\left[N_{i}\right]-1\right)\\ & =M\left({\left(\frac{\sum_{i=1}^{M}N_{i}}{M}\right)}^{2}+V\left[N_{i}\right]-1\right)\\ & =\frac{{\left(\sum_{i=1}^{M}N_{i}\right)}^{2}}{M}-M+MV\left[N_{i}\right]\\ & =\frac{N^{2}}{M}-M+MV\left[N_{i}\right], \end{array}$$

where the third equality is valid because $E\left[N_{i}\right]=\frac{\sum_{i=1}^{M}N_{i}}{M}$. The minimum value of O is $\frac{N^{2}}{M}-M$, which is achieved when $V[N_{i}]=0$ because $V[N_{i}]$ is non-negative. This completes the proof of Theorem 1. □

**Remark 1.**  *For M XC groups with N clients in total, the total number of XOR operations decreases as the variance of the cardinalities of XC groups decreases.*

In this paper, we placed a higher priority on MC over XC to reduce the computational complexity for XC grouping and XOR coding by decreasing the number of candidate clients of XC without increasing the number of required transmissions. Finally, all the remaining clients, given by $G_{\mathrm{UC}}=U\backslash G_{\mathrm{LC}}\backslash G_{\mathrm{MC}}\backslash G_{\mathrm{XC}}$, will receive their videos through UC. The number of transmissions required for UC is calculated by $K_{\mathrm{UC}}=\left|G_{\mathrm{UC}}\right|$.

## 4. Proposed Cache Update Scheme Using Reinforcement Learning

In this section, we formulate a cache management problem for XOR coding-based streaming systems and propose a new cache update scheme using reinforcement learning to improve the efficiency of video streaming. In our problem, each client updates its cache by replacing a content stored in $C_{n}$ with $r_{n}$ after playing $r_{n}$.

**Figure 3:**
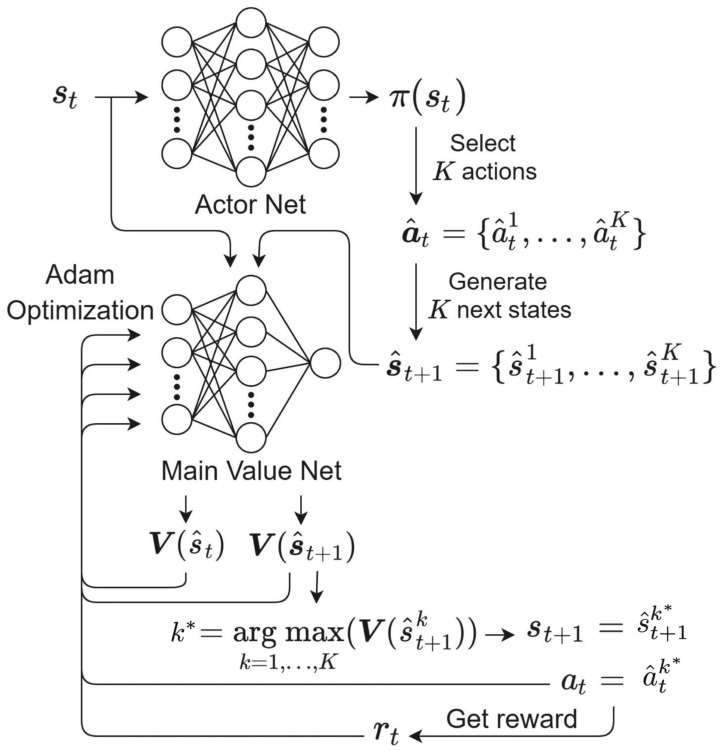
The proposed architecture of the *K*-AC.

**Figure 4:**
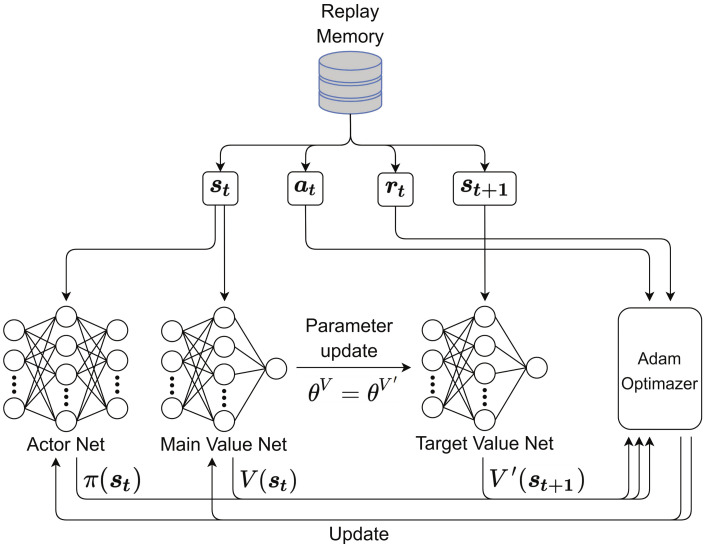
Illustration of the training process of the *K*-AC.

In conventional actor-critic (AC) networks, one action is only generated by actor networks, and the action may not be thus optimal with a high probability; it is also difficult to evaluate the value of the action generated by the actor network. In this paper, we thus proposed the *K*-actor-critic (*K*-AC) network to overcome the disadvantage of AC networks, which is depicted in Figure 3. The *K*-AC exists in each and every client and consists of an actor network and the main value network. $s_{t}$ and $\pi (s_{t})$ denote the input state and the output of actor network, respectively. $s_{t}$ for the client *n*, denoted by $s_{t}^{n}$, consists of 2(C+1) elements and is given as:

(15) $$\begin{array}{ccc} s_{t}^{n} & = & \{f_{t,s}^{n}\left(r_{n}\right),f_{t,s}^{n}\left(C_{n}\left(1\right)\right),\ldots ,f_{t,s}^{n}\left(C_{n}\left(C\right)\right),\ldots \\ \; & \; & f_{t,l}^{n}\left(r_{n}\right),f_{t,l}^{n}\left(C_{n}\left(1\right)\right),\ldots ,f_{t,l}^{n}\left(C_{n}\left(C\right)\right)\}, \end{array}$$

where $f_{t,x\in \{s,l\}}^{n}\left(v\right)$ denotes the view count of the video *v* for the client *n* during the last $L_{x\in \{s,l\}}$ video view times and $f_{t,s}^{n}\left(v\right)\leq L_{s}$, $f_{t,l}^{n}\left(v\right)\leq L_{l}$. $f_{t,s}^{n}\left(v\right)$ and $f_{t,l}^{n}\left(v\right)$ represent the frequency of the video *v* for a short-term period and a long-term period, respectively; thus, $L_{s}<L_{l}$. Each client updates its cache by replacing one video stored in its cache with the requested video $r_{n}$ or keeps the cache as it is. Thus, $a_{t}$ denoting an action that each client can take is defined as $a_{t}\in A=\left\{0,1,2,\ldots ,C\right\}$. The video $C(a_{t})$ will be replaced by $r_{n}$ if $1\leq a_{t}\leq C$. $a_{t}=0$ denotes that the cache will be kept in its current state, which leads to |A|=C+1. The output $\pi (s_{t})$ has the same size as A. Contrary to conventional AC networks that choose a single action, the proposed *K*-AC selects the *K* elements with the largest value in $\pi (s_{t})$ as candidate actions, which are denoted by ${\hat{a}}_{t}=\left\{{\hat{a}}_{t}^{k}|{\hat{a}}_{t}^{k}\in A,1\leq k\leq K\right\}$. If K=1, the *K*-AC becomes a conventional AC network. ${\hat{a}}_{t}$ generates the set of *K* next states ${\hat{s}}_{t+1}=\left\{{\hat{s}}_{t+1}^{k}|1\leq k\leq K\right\}$. The main value network evaluates the values of ${\hat{s}}_{t}$ and ${\hat{s}}_{t+1}$ by yielding $V({\hat{s}}_{t})$ and $V({\hat{s}}_{t+1})$, respectively, and the final action is selected as $a_{t}={\hat{a}}_{t}^{k^{*}}$, where $k^{*}=\underset{k\in \{1,\cdots ,K\}}{\arg\;\max\;V\left({\hat{s}}_{t+1}\right)}$, while the corresponding next state is determined by $s_{t+1}={\hat{s}}_{t+1}^{k^{*}}$. We designed rewards for our neural network in each client to minimize the number of transmissions per each client’s video view. The rewards for each client are defined as:

(16) $$r_{t}=\left\{\begin{array}{cc} 1 & \mathrm{for}\;\mathrm{LC}\\ 0.5 & \mathrm{for}\;\mathrm{MC}\;\mathrm{or}\;\mathrm{XC}\\ 0 & \mathrm{for}\;\mathrm{UC} \end{array}\right.,$$

where LC has the largest reward because it requires no video transmissions, MC and XC have the second largest and the same reward because they can reduce the number of video transmissions by sharing network resources with other clients, and UC has the lowest reward because it cannot reduce the number of video transmissions. The number of transmissions might be a better reward than that in (16) because our goal was to reduce the number of transmissions. However, the proposed learning model was designed to be trained and run in a fully distributed manner without information exchange with other devices or the server, and it is thus impossible for each client to know the final number of transmissions. We used a replay memory and the concept of mini batch to train our networks by updating the parameters of the actor and main value networks, as depicted in Figure 4. The size of the mini batch is *B*. Through a back propagation, the parameters of the main value network are updated first, and those of the actor network are then updated. The parameters of the main value network are trained by using the *B* random samples to minimize the loss, which is defined as:

(17) $$L_{V}:=\frac{1}{B}\sum_{i=1}^{B}{\left(r_{t}^{i}+\gamma \cdot V^{\prime }\left(s_{t+1}^{i}\right)-V\left(s_{t}^{i}\right)\right)}^{2},$$

where γ, denoting a discount factor, satisfies 0≤γ≤1 and $V^{\prime }\left(s_{t+1}^{i}\right)$ is the output of the target value network. The target value network is used to generate the target Q-values for computing the loss during training and to keep the network from being destabilized by falling into feedback loops between the target and estimated Q-values. The parameters of the target value network are fixed and periodically updated by being replaced by those of the main value network. The parameters of the main value network, $\theta^{V}$, are updated by the following gradient descent method:

(18) $$\theta^{V}\leftarrow \theta^{V}+\alpha \;\nabla_{\theta^{V}}L_{V},$$

where α denotes a learning rate. The loss function of the actor network is defined as:

(19) $$L_{A}\frac{1}{B}\sum_{i=1}^{B}\log\;\pi \left(a_{t}^{i}|s_{t}^{i}\right)A\left(s_{t},a_{t}\right),$$

where $A(s_{t},a_{t})$ denotes the advantage function of the actor network and can be calculated as:

(20) $$A\left(s_{t},a_{t}\right)=r_{t}+\gamma \cdot V^{\prime }\left(s_{t+1}\right)-V\left(s_{t}\right).$$

Finally, the parameters of the actor network $\theta^{\pi }$ are also updated by the gradient ascent method as follows:

(21) $$\theta^{\pi }\leftarrow \theta^{\pi }+\beta \;\nabla_{\theta^{\pi }}L_{A},$$

where β denotes a learning rate.

## 5. Numerical Results

In this section, we analyze the efficiency of the proposed cache update scheme using the *K*-AC in terms of the average number of transmissions per video streaming per client, which is defined as:

(22) $$\eta =E\left[\frac{K_{\mathrm{MC}}+K_{\mathrm{XC}}+K_{\mathrm{UC}}}{N}\right],$$

and compare it to that of conventional cache update schemes for both XC and non-XC. 0≤η≤1, where η=0 if all videos are transmitted through LC, while η=1 if videos are all transmitted through UC. In the *K*-AC, the actor network consists of input, hidden, and output layers of sizes 2(C+1), 4(C+1), and (C+1), respectively. The hidden layer is fully connected with the input and output layers. The ReLU and softmax functions are used as the activation functions for the input and hidden layers, respectively [24]. The value networks are the same as the actor network except that the output size is one. All parameters for the actor and value networks were initialized by He Uniform [25] and then updated iteratively by the Adam optimizer [26]. In our simulations, *B* and γ were set to 10 and 0.9, respectively, and $L_{s}$ and $L_{l}$ were set to 10 and 100, respectively. We compared the performance of the proposed *K*-AC with that of conventional cache update algorithms such as LRU, LFU, and FIFO, where it was assumed that K=10.

**Figure 5:**
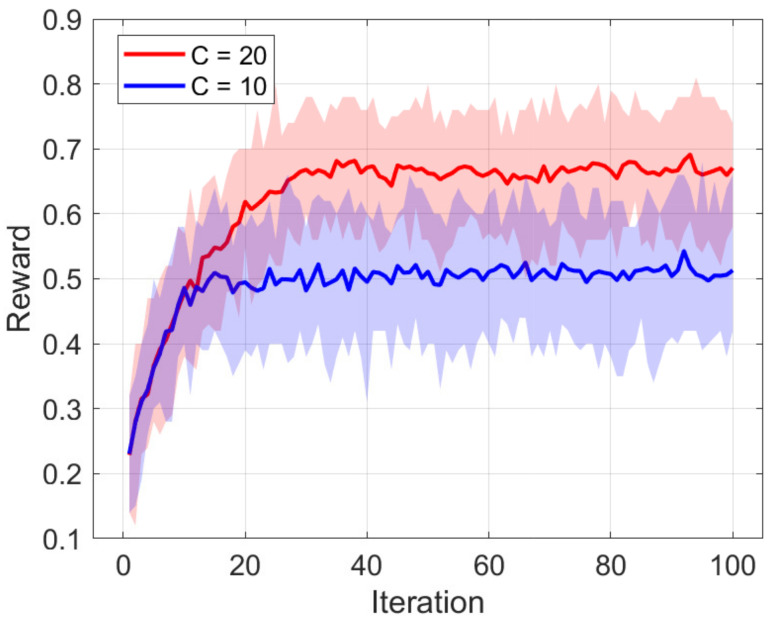
The rewards of the proposed scheme earned during a training process. p=0.001, ρ=0.5, V=100, N=50, K=10, and β=1.

Figure 5 shows the reward that the proposed *K*-AC scheme earns during a training process. *p*, denoting the probability that the popularity of videos changes, was set to 0.001, and the correlation factor ρ was set to 0.5. *V*, *N*, and β, denoting the number of videos, the number of clients, and the parameter of the Zipf distribution, were set to 100, 50, and 1, respectively. *C*, denoting the size of the cache, was set to 10 or 20. It is shown that the reward for C=10 stabilized faster than for C=20. More specifically, the reward for C=10 stabilized after about 20 iterations, whereas the reward for C=20 stabilized after about 40 iterations.

**Figure 6:**
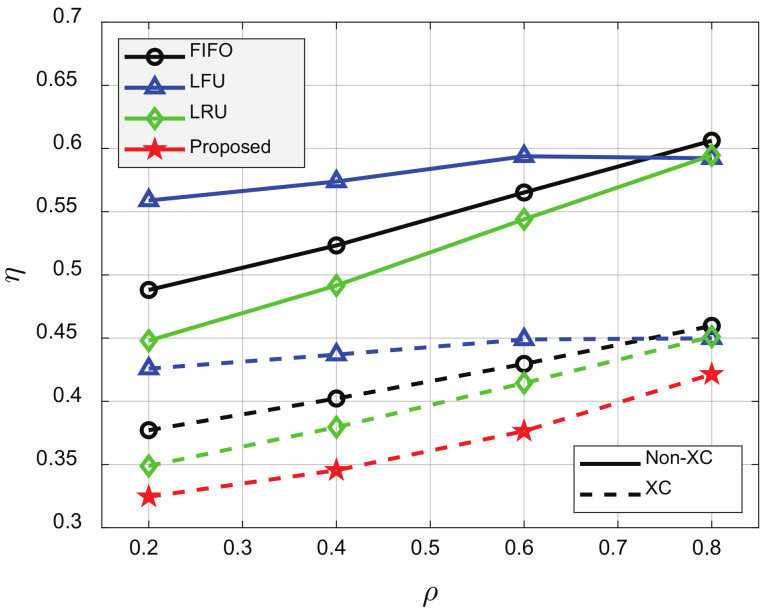
Average number of required transmissions for various ρ’s. p=0.001, V=100, N=50, C=20, K=10, and β=1.

**Figure 7:**
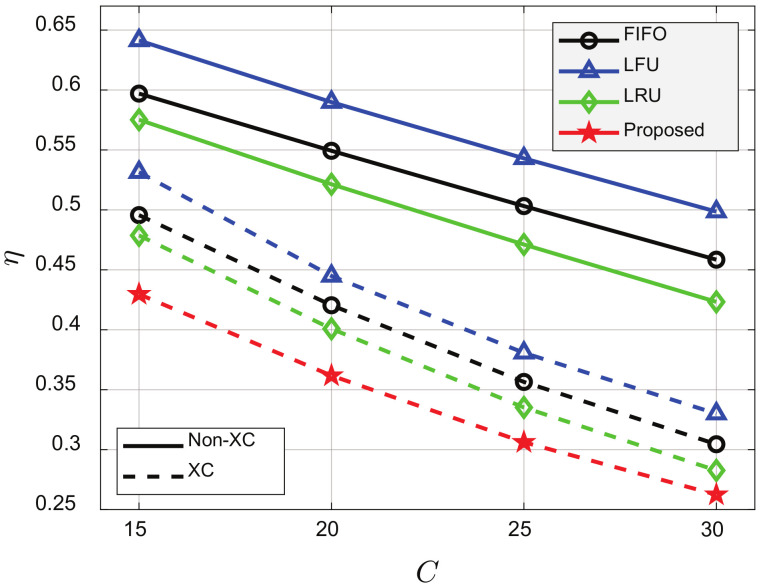
Average number of required transmissions for various *C*’s. p=0.001, ρ=0.5, V=100, N=50, K=10, and β=1.

**Figure 8:**
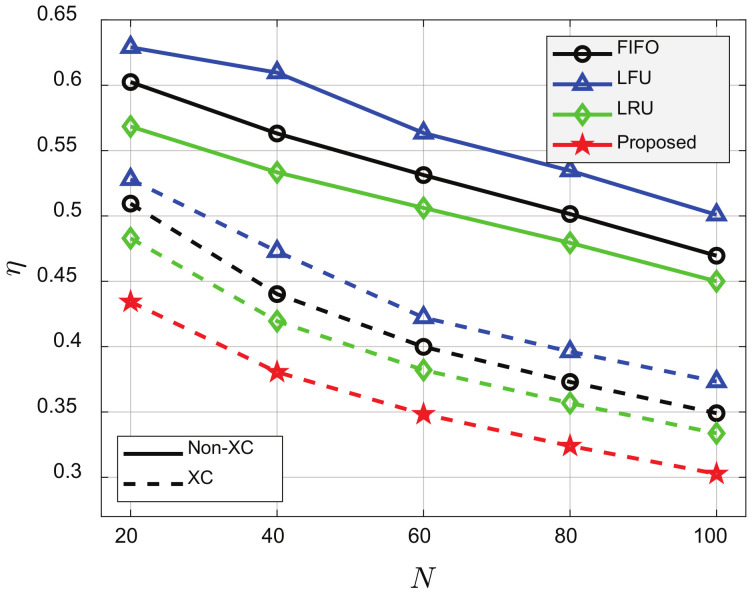
Average number of required transmissions for various *N*’s. p=0.001, ρ=0.5, V=100, C=20, K=10, and β=1.

**Figure 9:**
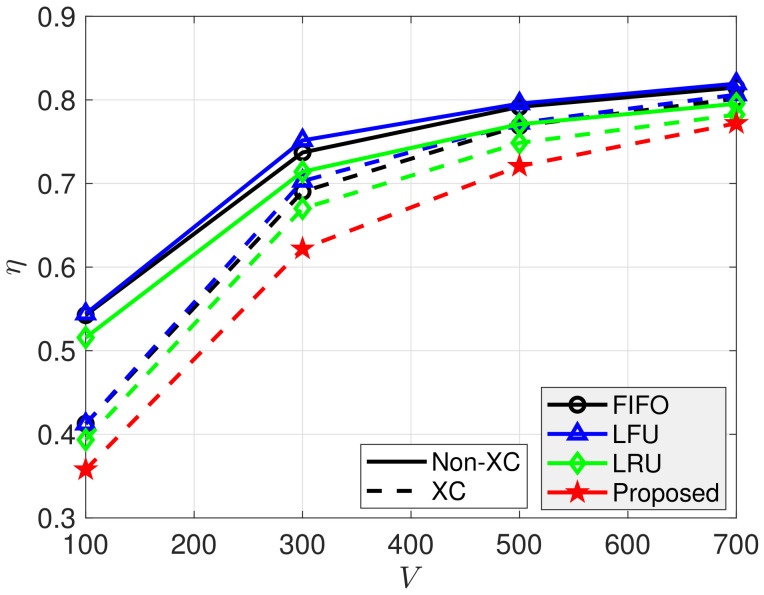
Average number of required transmissions for various *V* values. p=0.001, ρ=0.5, β=1, N=50, C=20, and K=10.

**Figure 10:**
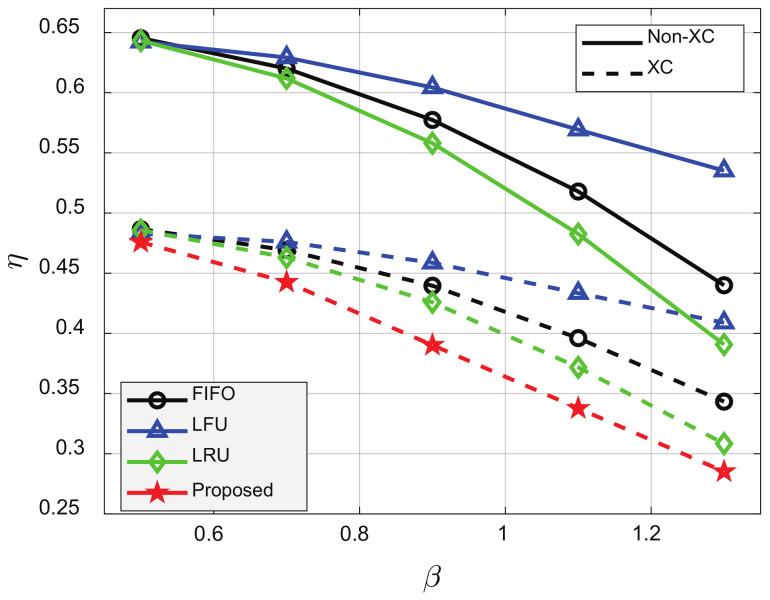
Average number of required transmissions for various β’s. p=0.001, ρ=0.5, V=100, N=50, C=20, and K=10.

Figure 6, Figure 7, Figure 8, Figure 9 and Figure 10 show the average number of required transmissions per view of the video per client, defined in (22), for ρ, *C*, *N*, and β, respectively. According to Figure 6, the XC video stream scheme outperformed the non-XC scheme regardless of the cache update algorithms. The non-XC scheme denotes the conventional video streaming with UC and MC without supporting XC. As ρ decreased, videos’ popularity changed less, and the average number of transmissions required for each video streaming decreased for all schemes. The proposed cache update scheme outperformed all conventional cache update schemes regardless of the value of ρ. For ρ=0.6, the XC video stream scheme reduced η by about 23.2%, 23.7%, and 23%, compared to FIFO, LFU, and LRU, respectively. In addition, the proposed cache update scheme could reduce η by about 8.8%, compared to LRU, which showed the best performance among the conventional schemes.

Figure 7 and Figure 8 show that η decreased as *C* or *N* increased for all schemes. The greater the *C*, the more the LC was because the probability that requested videos were already cached in the clients’ cache increased. The greater the *N*, the more the MC or XC was where multiple videos can be transmitted by single transmission. In addition, the XC video streaming scheme outperformed the non-XC video streaming scheme for all cache update schemes, and the proposed cache update scheme based on *K*-AC yielded the best performance. In Figure 7, when C=15, the XC video streaming scheme could reduce η by about 16.5%, 16.7%, and 16.3%, compared to FIFO, LFU, and LRU, respectively, and the proposed cache update scheme could reduce η by about 9.9% compared to LRU, which yielded the best performance among the conventional schemes. In Figure 8, when N=20, the XC video streaming scheme could reduce η by about 18.6%, 15.6%, and 14.6%, compared to FIFO, LFU, and LRU, respectively, and the proposed cache update scheme could reduce η by about 9.7%, compared to LRU.

Figure 9 shows η for various *V* values. For constant *C* and *N*, the possibility of MC and XC decreased as *V* increased, and η thus decreased for all schemes as *V* increased. The proposed cache update scheme outperformed all conventional schemes for all *V* values. Finally, Figure 10 shows that η decreased as β increased because clients were inclined to request highly popular videos, and the probability of LC, MC, or XC also increased. For β=0.9, the XC video streaming scheme reduced η by about 23.1%, 23.4%, and 22.9%, compared to FIFO, LFU, and LRU, respectively, and the proposed cache update scheme could reduce η by about 8%, compared to LRU, which showed the best performance among the conventional schemes.

## 6. Conclusions

In this work, we investigated a cache management problem for XC video streaming systems, where each client needs to update its cache so as to increase the probability of XC with other clients, as well as its own hit probability, while each client’s hit probability has been only considered in conventional video streaming systems. We formulated a cache management problem for XC video streaming systems and investigated how to minimize the number of XOR operations. We also proposed how to update the clients’ cache to improve the efficiency of video streaming by decreasing the number of transmissions. Contrary to most existing studies assuming that all clients have the same popularity of videos and the popularity is time invariant, our study considered that the popularity varies over time and is differently distributed for each client. Based on these practical assumptions, we proposed a new cache update scheme using reinforcement learning. The proposed scheme used the *K*-AC network to overcome the disadvantages of conventional AC networks. Each client can train its own *K*-AC network by using the local information, which does not require any feedback or signaling, and can decide whether to update its cache. If a client decides to update its cache, the video to be replaced by a new one is decided by the action of the *K*-AC. Thus, the proposed scheme is completely decentralized. We analyzed the performance of the proposed scheme in terms of the average number of required transmissions per each video streaming per client, which was compared to that of conventional cache update schemes such as FIFO, LFU, and LRU. Our numerical results showed that XC video streaming outperformed non-XC video streaming, and the proposed cache update scheme using the *K*-AC yielded the best performance. Specifically, when V=100, N=50, C=15, and β=1, the ρ’s for non-XC LRU, XC LRU, and the proposed scheme were 0.58, 0.48, and 0.44, respectively. Thus, it can be concluded that the proposed scheme could reduce the number of transmissions by 24.1% and 8.3%, compared to the non-XC LRU and XC-LRU schemes, respectively.

## Data Availability

Not applicable.
